# Femtosecond laser fabrication of silver nanostructures on glass for surface enhanced Raman spectroscopy

**DOI:** 10.1038/s41598-019-53328-6

**Published:** 2019-11-19

**Authors:** Mark MacKenzie, Haonan Chi, Manoj Varma, Parama Pal, Ajoy Kar, Lynn Paterson

**Affiliations:** 1 Institute of Photonics and Quantum Sciences, School of Engineering and Physical Sciences, Heriot Watt University, Edinburgh, EH14 4AS UK; 2 Institute of Biological Chemistry, Biophysics and Bioengineering, School of Engineering and Physical Sciences, Heriot Watt University, Edinburgh, EH14 4AS UK; 3 Centre for Nano Science and Engineering (CeNSE), Indian Institute of Science, Bangalore, Karnataka India; 4 TCS Research and Innovation, Tata Consultancy Services, Bangalore, India

**Keywords:** Biomedical engineering, Optics and photonics, Laser material processing, Raman spectroscopy

## Abstract

We report on an optimized fabrication protocol for obtaining silver nanoparticles on fused silica substrates via laser photoreduction of a silver salt solution. We find that multiple scans of the laser over the surface leads to a more uniform coverage of densely packed silver nanoparticles of approximately 50 nm diameter on the fused silica surface. Our substrates yield Raman enhancement factors of the order of 10^11^ of the signal detected from crystal violet. We use a theoretical model based on scanning electron microscope (SEM) images of our substrates to explain our experimental results. We also demonstrate how our technique can be extended to embedding silver nanoparticles in buried microfluidic channels in glass. The *in situ* laser inscription of silver nanoparticles on a laser machined, sub-surface, microfluidic channel wall within bulk glass paves the way for developing 3D, monolithic, fused silica surface enhance Raman spectroscopy (SERS) microfluidic sensing devices.

## Introduction

Surface enhanced Raman spectroscopy (SERS) is a unique spectroscopic fingerprinting technique that stems from the interaction of molecules in close proximity to metallic nanoparticles. Since its discovery in the late 1970s^1^, it has been widely employed as a versatile, powerful, non-destructive bioanalytical tool owing to its inherent high detection sensitivities as well as chemical specificities. This renders it as ideal for label-free detection of trace analytes in multi-component solutions.

SERS is essentially the dramatic enhancement (up to six or more orders of magnitude) of Raman scattered signals from target molecules placed in the vicinity of nanostructured morphologies or colloidal nanoparticles due to two primary mechanism: chemical enhancement^2^ and electromagnetic enhancement^3^. The chemical enhancement mechanism for SERS is due to charge transfer between the analyte molecule and metallic nanoparticle. This is a short-range mechanism and only occurs when an analyte is absorbed to a nanoparticle surface^4^. The electromagnetic enhancement mechanism is due to light, in or nearby nanostructured electrically conducting materials (such as silver or gold), exciting conduction band electrons^5^. The electrons undergo coherent oscillations, so-called localized surface plasmon resonances (LSPRs)^6,7^. This localised electromagnetic field intensification near the nanoparticle surface, produced by incident light excitation of the appropriate wavelength and polarization, results in enhancement of the spectroscopic signal from molecules at or near the surface. This coupling of the incident electromagnetic field and LSPR decays exponentially with particle distance, but is known as a long-range mechanism, since it can extend to a distance two and a half times the nanoparticle diameter^8,9^. The LSPR bands for silver nanoparticles are within the visible part of the electromagnetic spectrum and expected to have a peak between 400–500 nm^10^. The LSPR of spherical 50 nm silver nanoparticles is around 430 nm^11,12^ with the location of the LSPR band highly dependent on the material, size, shape, aggregation state and surrounding material^11,13^. Increasing particle size results in a red shift of the LSPR peak^14^. High enhancement factors are achieved if the LSPR comes close to or overlaps with the incident light or the Raman lines^15,16^.

A critical aspect in designing sensor devices based on SERS is the enhancement factor (EF) which depends on several factors, including (but not limited to) the excitation wavelengths, nanostructure morphologies and dimensions, types of metals and the surrounding chemical environment. Traditionally, noble metals such as silver, gold and copper with nanoscale features are used as plasmonic substrates for observing SERS. However, fabricating stable, reproducible, position-controlled micro- and nano-structured components which can yield controllable EFs continues to be challenging experimentally. In this paper, we conduct a parametric study to determine optimal fabrication conditions for controlling the shapes and spatial uniformities of silver nanoparticles (AgNPs) obtained via a laser-induced photoreduction method. Using ultrafast laser writing technique we can produce uniform surfaces of AgNP on glass. We also describe the fabrication of AgNPs inside an embedded channel within glass. The same laser system to inscribe the microfluidic circuitry is also used to write the AgNP SERS sensor surface thus making SERS possible inside fabricated structures.

The preparation of colloidal AgNPs for use as a SERS substrate is relatively straightforward. Ag^+^ cations, provided by silver nitrate, are reduced using a chemical reducing agent, for example trisodium citrate, NaBH_4_, hydrazine or hydroxylamine hydrochloride^17–20^ producing colloidal suspensions of nanosilver. The subsequent concentration of AgNPs by centrifugation, addition of various molecules for modifications and surfactants to enhance shelf life, can be laborious and expensive^21^. Colloidal silver nanoparticles (AgNPs) display several disadvantages which limit their applications to spectroscopy, such as residual impurities in the suspension^22^, degradation^23^ and reproducibility of SERS measurements since analyte molecules must be located within ‘hot spots’ formed by nanoparticle aggregates. This can yield high enhancements, but are random and transitory^21,24,25^. Reproducibility of the shape, size, and size distribution of particles is greatly affected by both the temperature and the reducing agents of the reaction, with higher temperatures speeding up nanoparticle formation as well as improving monodispersity of silver colloids but as the heating duration is increased, aggregation and grain growth is observed^26–28^.

In an effort to fabricate SERS substrates with high sensitivity, signal uniformity, and reproducibility, SERS-active surfaces on glass slides and silicon wafers have been made by depositing colloidal nano-silver dispersed in appropriate solvents on the substrates, but led to disperse coverage resulting in low reproducibility^22^ and limited electromagnetic field enhancement^29^. Other methods to generate or immobilise metallic nanoparticles on solid support substrates include self- assembly^30–32^, chemical surface patterning techniques^33^, template-based methods^34^ and nanolithography, including electron-beam (e-beam) lithography^35,36^, focused ion beam (FIB) milling^37–39^ and nanoimprinting^40^. AgNPs have been deposited onto nanowire structures^41–43^ or the active SERS area^44^. Recent reports describe the fabrication of paper-based SERS devices. AgNPs were drop-casted onto hydrophilic wells patterned on different types of paper^45^. The device’s potential suffers from reduced sensitivity and specificity due to large background Raman signals from the filter paper material and the dispersion of nanoparticles through the porous material. Less porous office paper was favourably found to result in a more concentrated layer of AgNPs and less background signal. However, the deposition of AgNPs is not well controlled when dropped onto paper^45,46^. Alternative methods to deposit metal NPs on paper substrates for SERS are inkjet printing of colloidal solutions^47^, printing silver salt solutions for development into SERS substrate at the time of use^21^, laser techniques^48^ and deposition of AuNPs onto nanopillars or wires grown or printed on the paper^49^.

Colloidal AgNPs have been generated using UV photoreduction of silver nitrate without a chemical reducing agent such as sodium citrate^50^ and more recently femtosecond lasers were used to write AgNPs on glass using silver nitrate and sodium citrate in the precursor sample^51^ and another study with no reducing agent and silver nitrate only in the precursor sample^52^. It was hypothesised that the AgNPs are made via multiphoton absorption of the pulsed beam by the precursor solution resulting in photoreduction of the Ag^+^ ions^51^. It was suggested that a higher concentration of silver nitrate solution is required to increase the population of nanoparticles^52^ and laser scanning speed should be slow enough to allow the photoreduction to occur and AgNPs to form^52^. In another study a thin layer of silver nitrate on a silicon wafer has been photoreduced by femtosecond laser without the addition of sodium citrate to create a SERS substrate^53^. It was observed that too high a laser power results in the removal of the thin layer leading to poor coverage of AgNP. The role of both chemical reduction and photoreduction on the shape, size and spacing of the AgNPs, produced in colloidal solutions or immobilised on solid supports, remains to be elucidated.

Fused silica is an excellent candidate for SERS substrates owing to its minimal Raman signal^54^. This aspect, coupled with ultrafast laser inscription techniques for writing reproducible, sub-wavelength structures directly using femtosecond laser pulses by virtue of strong, highly localized nonlinear optical effects^55^, enables a unique approach for obtaining versatile SERS-active devices at high throughputs. This direct laser writing technique can be used to write on surfaces and also to create sub-surface structures due to the multiphoton effect at the beam focus only^56^. In this paper we use femtosecond laser pulses to photo-reduce silver cations and write SERS AgNP surfaces on fused silica substrates in a controllable manner. We discuss the writing parameters used, the surfaces created and their correlations with measured SERS enhancements.

## Materials and Methods

### Fabrication of SERS structures

**Figure 1:**
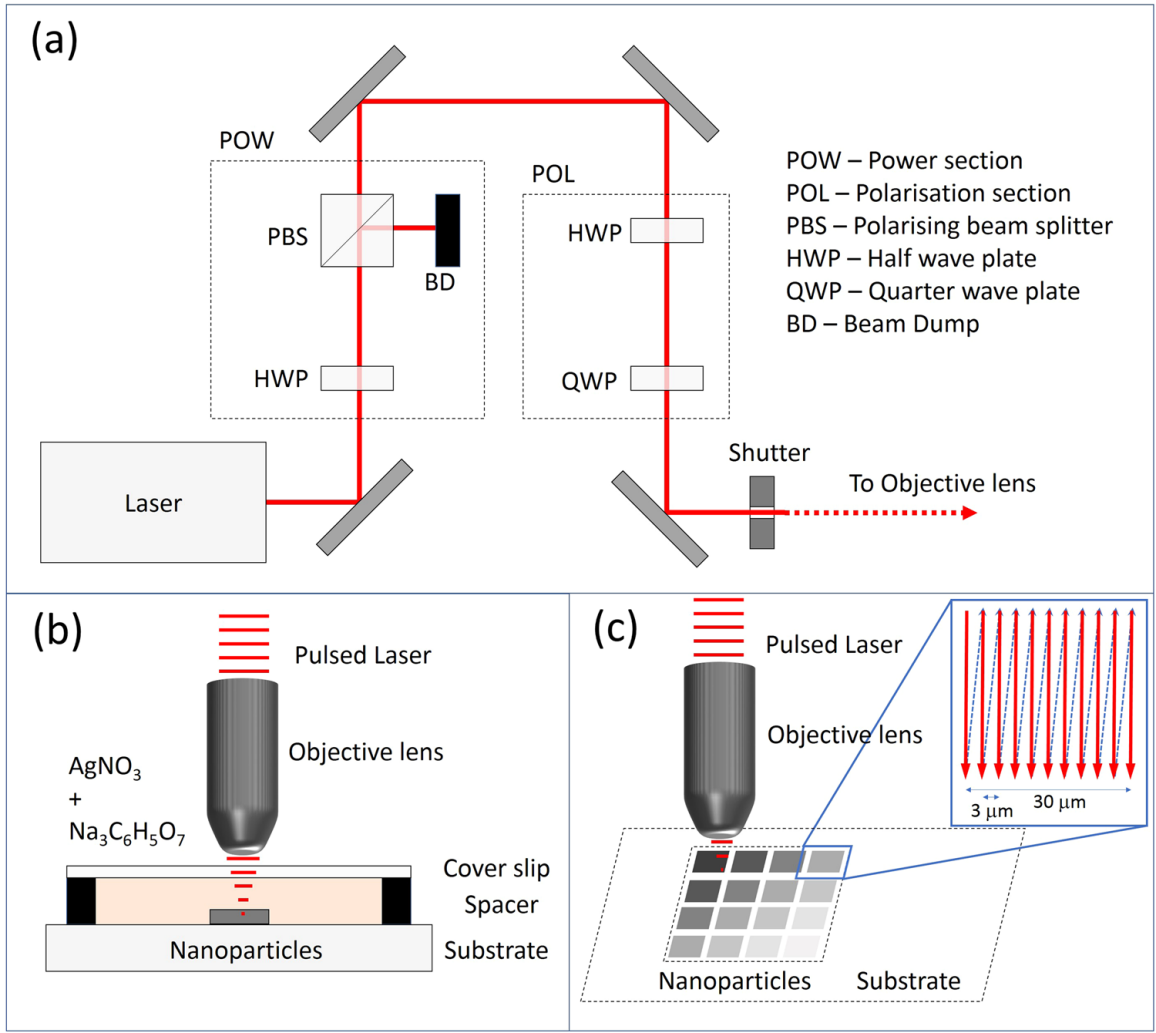
Schematic of writing process; (**a**) laser writing system, (**b**) laser fabrication of nano-colloid on the chip, (**c**) inscription pattern.

The setup for writing the SERS substrates comprised of an IMRA μ-Jewel Ytterbium fibre laser operating at 1047 nm (500 kHz repetition rate, 350 fs pulse width) as the femtosecond laser excitation source for writing the AgNP structures. A 0.4 numerical aperture (NA) microscope objective was used to focus the laser beam onto the target surface. Fused silica substrates were placed on software-controlled, high-precision horizontal and vertical stages (Aerotech ABL10100 and Aerotech AVL125, respectively) that operated with a repeatability of 50 nm in horizontal and 300 nm in vertical direction. The computer was also used to control the laser power and polarization state of the incident beam through wave plates and polarizers. A charged coupled device (CCD) camera was used to monitor the fabrication process in real-time. The setup can be seen in Fig. 1a. A schematic of the writing region is shown in Fig. 1b. Silver nanoparticles were generated *in-situ* by photo-irradiating a 1:3 mixture of 1 mM sodium citrate solution (Na_3_C_6_H_5_O_7_; C8532, Sigma Aldrich) and 1 mM silver nitrate solution (AgNO_3_; 35375, Sigma Aldrich). 20 µl of the precursor mixture was pipetted into a round, 10 mm diameter, 80 *μ*m high chamber created on the fused silica using a vinyl spacer. A glass coverslip was placed on top. The laser was focused through the glass coverslip onto the surface of the fused silica. Initially, a ‘damage’ line was written on the fused silica surface using a laser power of 200 mW to mark the location of the SERS region, enabling it to be easily found later for the Raman measurements. Using a lower laser power, of no more than 30 mW, photo-reduction of the silver salt solution was induced by the laser, resulting in the creation of colloidal silver nanoparticles which adhered to the fused silica surface. Multiphoton absorption by the precursor solution results in the photoreduction of the Ag^+^ ions, and due to the presence of sodium citrate in the sample, there is a degree of chemical reduction too. The femtosecond laser also inscribes the AgNPs onto the fused silica surface. The substrate was translated through the focused laser beam such that eleven closely-spaced parallel lines, spaced 3 *μ*m apart, are written on the fused silica, creating a square pattern of approximately 30 *μ*m length, as shown in Fig. 1c.

A parametric study was performed to determine the optimal fabrication conditions for generating SERS substrates. Laser powers of 5, 10, 20 and 30 mW and stage scan speeds (0.1, 0.2, 0.4 and 0.6 *μ*m s^−1^) were used to inscribe SERS structures. The number of scans was also investigated. Laser powers of 30 mW or greater were not used due to the generation of gas bubbles due to thermal effects, which impede the fabrication by interrupting the beam path. After inscription of the SERS substrate is complete, the substrates are air-dried for subsequent scanning electron microscope (SEM) analysis and for use in SERS sensing experiments. Optical images of the inscribed surfaces were taken using a Leitz Ergolux microscope and SEM images taken with FEI Quanta 3D FEG Dual beam SEM/FIB. Results of single scans and the multiscan technique are presented later.

### Raman microspectroscopy

All Raman spectra were acquired using a Raman microspectrometer (inVia, Renishaw plc) equipped with a Peltier cooled charge-coupled device camera for spectral detection and a 514 nm laser for Raman excitation. A Leica 50× objective was used to collect the Raman signals from the sample unless otherwise stated. Samples were illuminated using between 2–20  mW of 514 nm laser light. Each spectrum was typically acquired using a 1 to 10 second exposure time and with a spectral resolution of 2 cm^−1^ in the wave number range of 100–1,000 cm^−1^. The spectrograph was calibrated before experimental sessions using the 521 cm^−1^ Raman line of a Si wafer. Several spectra from analytes were measured from different locations on the silver surface for each sample to obtain a mean spectrum from the substrate or to understand the variability of enhancement within a specific inscribed area. All spectra were recorded at room temperature. The software package WIRE 3.0 (Renishaw plc) was used for spectral acquisition.

The analytes used in this study were Rhodamine 6G (R6G) (R4127, Sigma Aldrich) and crystal violet (CV) (C3886, Sigma Aldrich), which were dissolved in water to yield solutions of varying concentrations. Twenty µl of analyte was added drop-wise into the 10 mm diameter sample chamber, onto the multiple 30 *μ*m^2^ femtosecond laser written SERS substrates, left undisturbed for ten minutes at room temperature and then air dried before Raman enhanced spectra were acquired from SERS surfaces and from fused silica which had not been modified by the laser. The intensity of peak in the crystal violet (CV) signature at 915 cm^−1^ was used to calculate the average analytical SERS enhancement factor (AEF) with a limit of 200 counts used as a threshold for a detectable SERS signal.

The Raman scattering enhancement factor (EF) can be estimated using the following equation:

1 $$EF=\frac{{I}_{SERS}{N}_{R}}{{I}_{R}{N}_{SERS}}$$

where, *I*_*SERS*_ is the SERS intensity (as measured on the AgNP surface); *I*_*R*_ is the standard Raman intensity (not enhanced, measured on a fused silica surface); *N*_*SERS*_ and *N*_*R*_ represent the number of molecules probed on the SERS surface and in the scattering volume on a non-SERS surface, respectively. Since we do not know the number of molecules probed and wish to know how much more signal is generated by SERS compared to normal Raman, the analytical enhancement factor (AEF) as described by Le Ru *et al*.^57^ (also known as the apparent enhancement factor)^58^ can be calculated using the following definition:

2 $$AEF=\frac{{I}_{SERS}{C}_{R}}{{I}_{R}{C}_{SERS}}$$

where *C*_*R*_ is the concentration of an analyte which produces a Raman signal, of intensity *I*_*R*_, under non-SERS conditions. *C*_*SERS*_ is the concentration of the same analyte which gives a SERS signal, of intensity *I*_*SERS*_, under identical experimental conditions (Raman spectrometer, microscope objective lens, excitation laser wavelength, power and exposure time). An extension of this, if different laser powers and accumulation times are used to generate the Raman signals is Eq. (3)^53,59^,

3 $$AEF=\frac{{I}_{SERS}{C}_{R}{P}_{R}{t}_{R}}{{I}_{R}{C}_{SERS}{P}_{SERS}{t}_{SERS}}$$

where *P*_*R*_ is the laser power used to generate the standard Raman signal from the analyte (mW), *t*_*R*_ is the accumulation time (s) for the standard Raman signal, *P*_*SERS*_ is the laser power used to acquire the SERS signal (mW) and *t*_*SERS*_ is the accumulation time for the SERS signal. An alternative way to express this is

4 $$AEF=\frac{[{I}_{SERS}/{P}_{SERS}{t}_{SERS}]/{C}_{SERS}}{[{I}_{R}/{P}_{R}{t}_{R}]/{C}_{R}}$$

where ${I}_{SERS}/{P}_{SERS}{t}_{SERS}$ and ${I}_{R}/{P}_{R}{t}_{R}$ are in units of counts mW^−1^ s^−1^. We have used Eq. 4 to perform our AEF calculations.

To determine uniformity of the enhancement factor across the 2D written SERS surface, Raman mapping was performed using a scan step size of 1.5 *μ*m across a 64 × 64 *μ*m area.

## Results and Discussion

### Single scan

**Figure 2:**
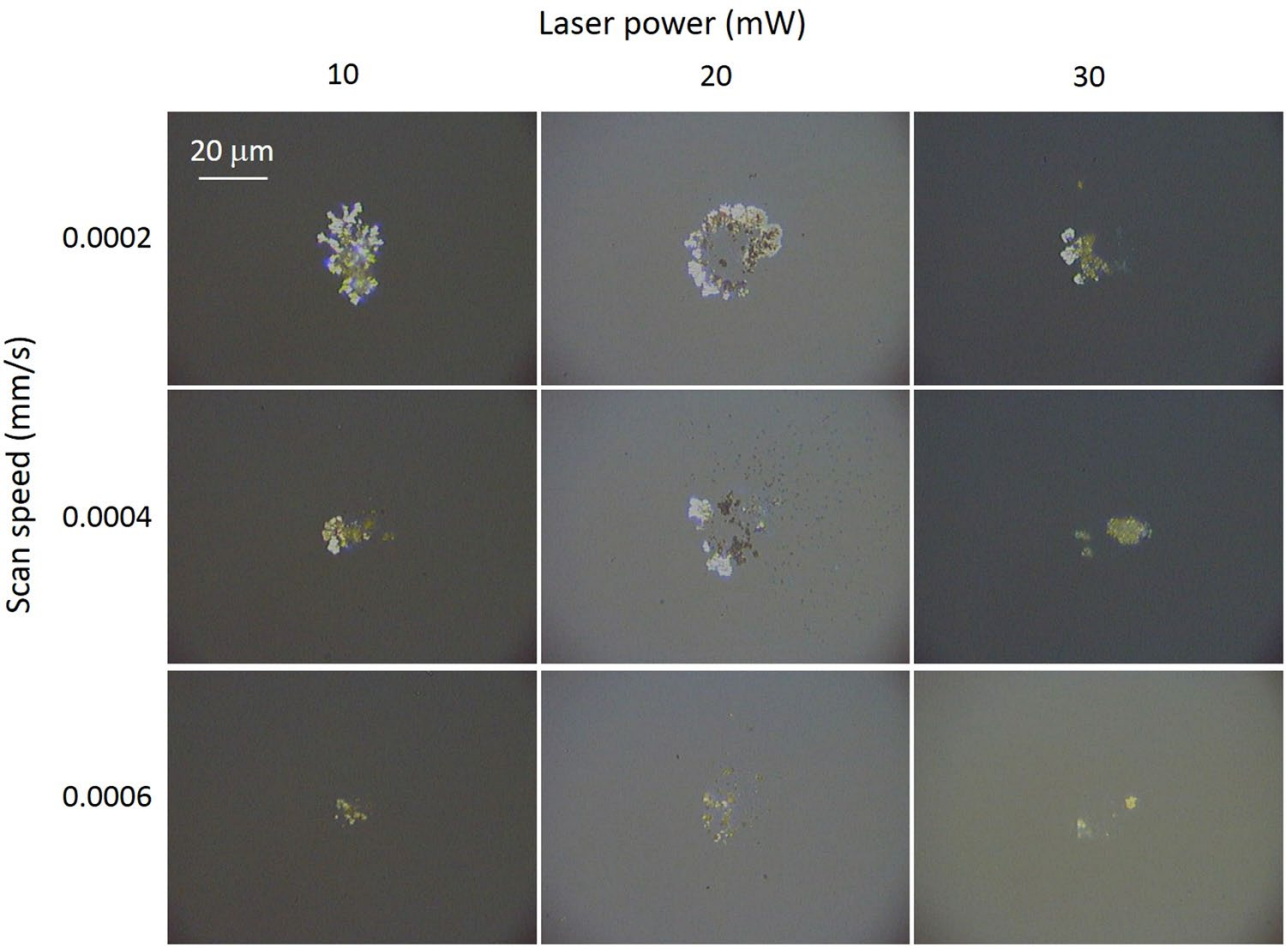
Optical images of inscribed surfaces using different laser inscription powers and stage scan speeds.

Initially, nanopatterned regions were fabricated using a single scan technique using the parameters mentioned in the previous section. Figure 2 shows representative optical images of these regions, with laser scan speed and power shown. The total energy deposited on each area is calculated using (path length/scan speed) × laser power. The path length is 330 *μ*m for each area. From Fig. 2 it can be seen that for a given power, decreasing the inscription speed leads to an increase in the extent of the area covered with silver nanoparticles. However, for a given scan speed, increasing the laser power, and thus the energy deposited, does not appear to lead to an increase in the area of the written substrates.

**Figure 3:**
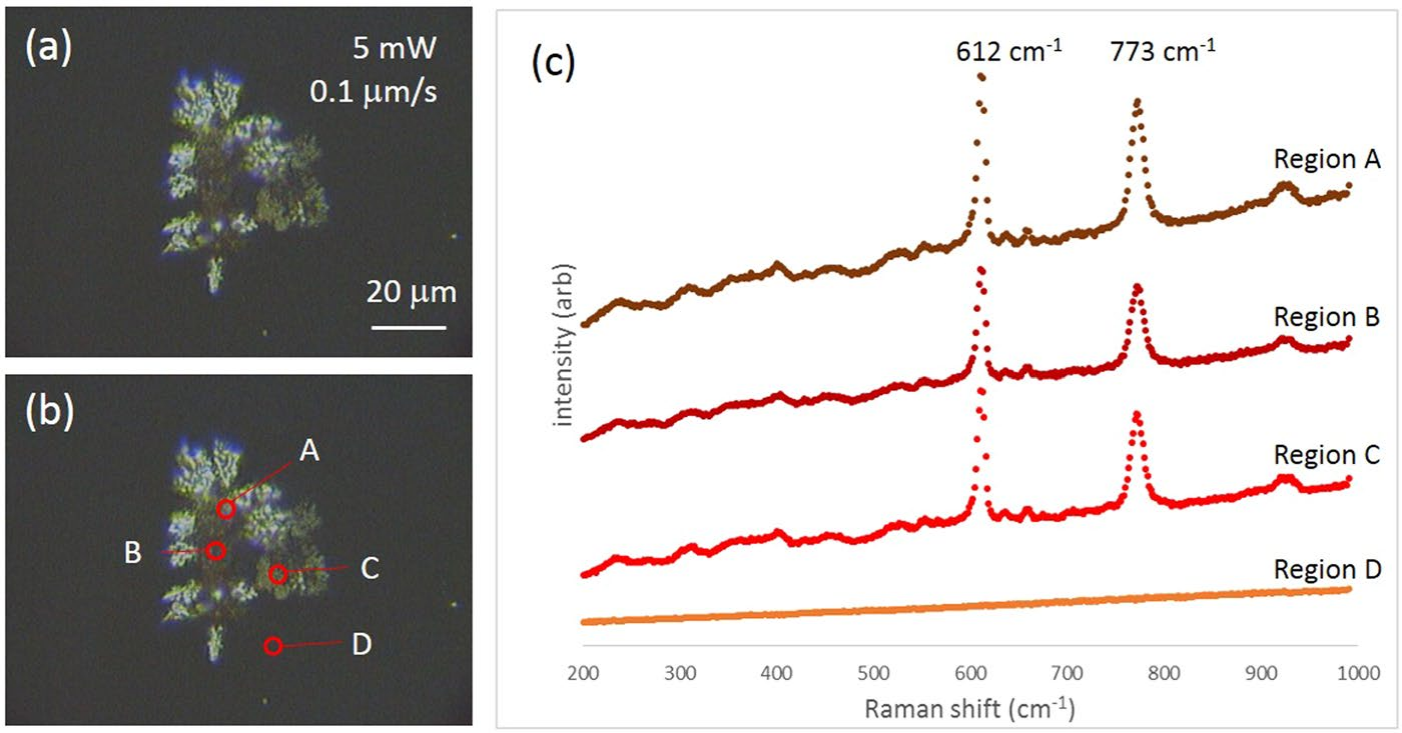
(**a**) Optical image of inscribed surface using 5 mW of laser writing power at a scan speed of 0.1 μm/s, (**b**) locations on surface where Raman spectra of R6G is acquired, (**c**) Raman spectra acquired from regions A, B, C and D.

Due to our observations that powers over 30 mW heat the samples such that bubbles are generated leading to poor substrate fabrication, and that lower writing speeds lead to better coverage of fused silica with the silver substrate, we subsequently used 5 mW of laser power and a scan speed of 0.0001 mm/s (energy deposition of 16.5 J) to photo-irradiate our substrates. This resulted in denser coverage interspersed with large gaps (where no nanoparticles were observed) within the inscription region, as seen in Fig. 3a. To determine if the substrate provided an enhancement to the Raman signal, 1 mM R6G was added to the substrate and multiple locations within the inscribed region were subsequently interrogated for Raman analysis. Figure 3b shows four regions where Raman spectra were collected from and Fig. 3c shows representative SERS enhancements of R6G molecules absorbed in the surface, from these regions.

Regions A, B and C in Fig. 3b were chosen as representative regions with AgNPs present and Raman spectra acquired from these three locations clearly show the Raman lines of R6G at 612 cm^−1^ and at 773 cm^−1^. Region D, representative of a region with no, or few, AgNPs shows no detectable R6G Raman signal. The uneven, patchy inscription of the silver substrate on the surface of fused silica fabricated using a single scan of the pattern in Fig. 1c with 5 mW of power and a writing speed of 0.1 *μ*m/s results in non-uniform enhancement of the Raman signal from R6G over the surface. A more uniform coverage of AgNPs is key to achieving consistent Raman enhancements implying that multiple scans over the target region, as opposed to a single scan, are required.

### Multi-scan SERS substrates

Similarly to the section above, 30 *μ*m × 30 *μ*m square SERS substrates were fabricated on the surface of fused silica, this time by writing over the squares multiple times.

**Figure 4:**
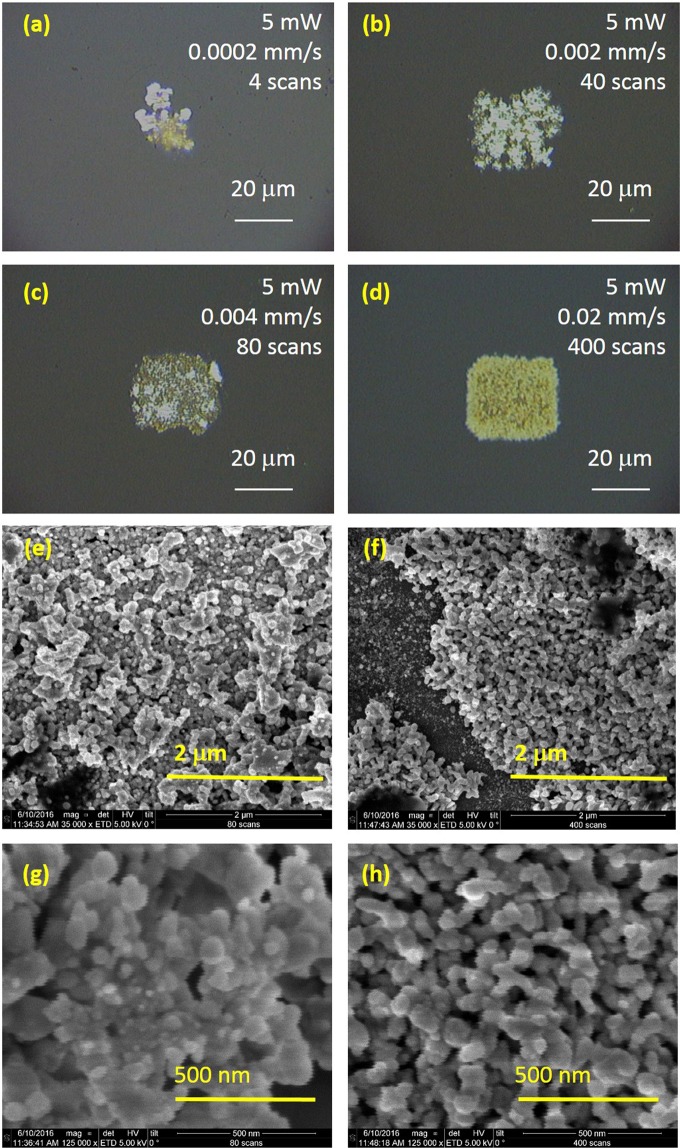
Optical images of AgNPs written with different numbers of scans. The laser writing power was 5 mW and the energy deposited was 33 J within the 30 × 30 um square. (**a**) 0.0002 mm/s scan speed, 4 scans, (**b**) 0.002 mm/s scan speed, 40 scans, (**c**) 0.004 mm/s, 80 scans, (**d**) 0.02 mm/s, 400 scans, (**e**) SEM image of surface made with 80 scans, (**f**) SEM image of surface made with 400 scans, (**g**) SEM image of surface made with 80 scans, (**h**) SEM image of surface made with 400 scans.

Figure 4 shows representative optical and SEM images of the SERS regions fabricated using these parameters. The laser power was fixed at 5 mW in all cases and the energy density maintained at 0.1 J/μm. The optical images Fig. 4a–d indicate a positive correlation between scan number and uniformity of SERS surface. They are noticeably more opaque than the single scan regions in Figs. 2 and 3 with more uniform coverage of AgNPs. The surface with 400 scans made with a writing speed of 20 *μ*m/s appears most uniform in the optical images. SEM images show some similarity in the opaque surfaces made by 80 scans (Fig. 4e,g) and 400 scans (Fig. 4f,h) of the pattern, with disordered arrays of connected nanosphere structures of approximately 50 nm in diameter.

**Figure 5:**
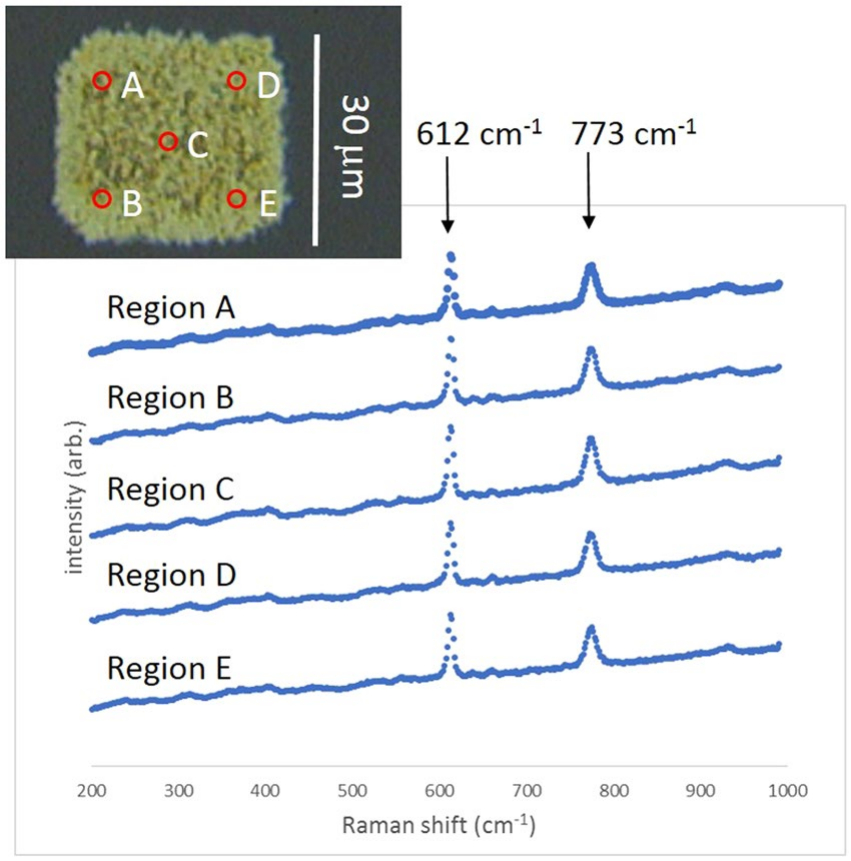
Raman spectra from 1 mM R6G collected from five locations on a multiscan SERS substrates.

Five locations on the surface shown in Fig. 4d and Fig. 5 (made using 400 scans at 20 *μ*m/s) were chosen to detect the SERS signal from 1 mM R6G to determine the intensity and uniformity of the signal. 2 mW of Raman excitation power, wavelength of 514 nm and 1 s exposure time was used. The raw spectra can be seen in Supplementary Fig. S1. The average peak height of R6G’s 612 cm^−1^ signal from regions A, B, D and E towards the corners of the AgNP region was around 9,500 counts with a relative standard deviation of 2.12% demonstrating excellent uniformity of this surface. The 612 cm^−1^ peak height from region C, within the centre of the region however, was higher, at approximately 10,800 counts, leading to an average peak height across the surface of 9838 counts +/− 5.81%. The average peak height for the 773 cm^−1^ line across the five locations was 6015+/− 7.05%, similar to that quoted for commercial substrates. The concentration of 1 mM R6G is notably high and the uniform signal may be due to saturation of the signal due to the large number of molecules present on the surface, so lower concentrations of analyte were used next.

The limit of detection (*C*_*SERS*_ in Eq. (4)) on our 400-scan SERS surface was calculated by detecting the 915 cm^−1^ peak of CV (or the 612 cm^−1^ peak of R6G) at a variety of concentrations using different Raman laser excitation powers. The peaks of 1 nM concentration analyte could be observed using 2 mW of laser power, 1 second accumulation. At 100 pM concentration the peaks were seen when using 20 mW laser power, 1 second accumulation. At 10 pM concentration, the peaks could only just be detected using 20 mW laser power, 1 second accumulation and at 1 pM the Raman peaks could no longer be observed using these parameters. Using higher laser power or longer accumulation time may have resolved the peak at 1 pM concentration, but there was a risk that the AgNPs could be damaged under those conditions. Thus we concluded that for the 400 scan surface, the limit of detection is 10^−11^ M for both R6G and CV.

The limit of detection for both analytes on fused silica (non-SERS) substrate (*C*_*R*_) was found to be 0.1 M, using 10 mW, 1 second accumulation. The average intensity, *I*_*R*_, of the CV 915 cm^−1^ peak from 8 regions on the non-SERS substrate, at a concentration of 0.1 M, was 260 counts. These spectra can be seen in Supplementary Fig. S2.

**Figure 6:**
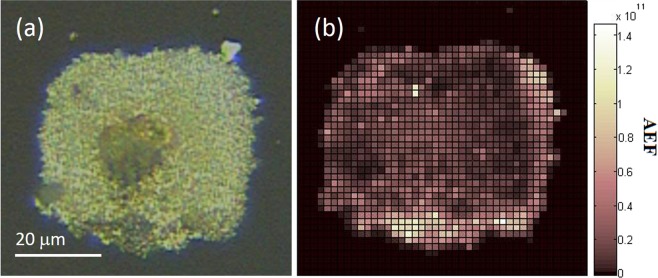
(**a**) Optical image of 400-scan substrate used for Raman mapping, (**b**) Raman map showing analytical enhancement factor (AEF) by measuring the intensity of the 915 cm^-1^ peak from 10^−11^ M crystal violet. Pixel size is 1.5 × 1.5 *μ*m.

A 2D Raman map was generated by scanning a 20 mW Raman excitation beam across the AgNP SERS surface in the presence of 10^−11^ M CV (*C*_*SERS*_) and recording the intensity of CV’s 915 cm^−1^ Raman peak, and a background signal at 883 cm^−1^, for each pixel. Accumulation time for each pixel was 1 s. These data can be seen in Supplementary Data. The 915 cm^−1^ line from CV on the AgNP surface was measured to be maximum of around 2000 counts depending on the region analysed. The average background of the 883 cm^−1^ line, not associated with CV, was measured to be 140 counts. The peak intensity from each pixel, minus the background gave a value for *I*_*SERS*_. Equation (4) was used to generate a heat map of analytical enhancement factor (AEF) across the surface (Fig. 6b). In Fig. 6b it can be seen that the analytical enhancement factors are not the same at the different regions, so the SERS response is not homogeneous across the AgNP surface. Of the total number of pixels in the image (44 × 44 = 1936), 72% of the area shown in the figure (1394 pixels) has AgNP present and an AEF of 10^6^ or above, the rest of the area is glass with no AgNP surface so no enhancement is observed in those areas. 0.5% of the pixels in the figure shows an AEF of >10^11^, 39% of the pixels have an AEF between 10^10^ and 10^11^, 13% have an AEF between 10^9^ and 10^11^ and 14% of the area of the figure have an AEF between 10^8^ and 10^9^. The average AEF across the AgNP surface is calculated to be 1.7 × 10^10^ with a large RSD of 118%, not uncommon when using low concentrations at the limit of detection.

We observe a good correlation between densely patterned regions in the brightfield microscope images of the substrate (Fig. 6a) and the AEF maps (Fig. 6b) thereby indicating a positive correlation between the enhancement factors and the nanoparticle density. The highest enhancement factor was determined to be 1.4 × 10^11^, similar to that of commercial substrates. Further work to determine the linear response of intensity to concentration, limit of detection and limit of quantification of this and other laser machined SERS substrates remains to be done before these devices can be used for molecular quantification studies.

### SERS-active buried channels

**Figure 7:**
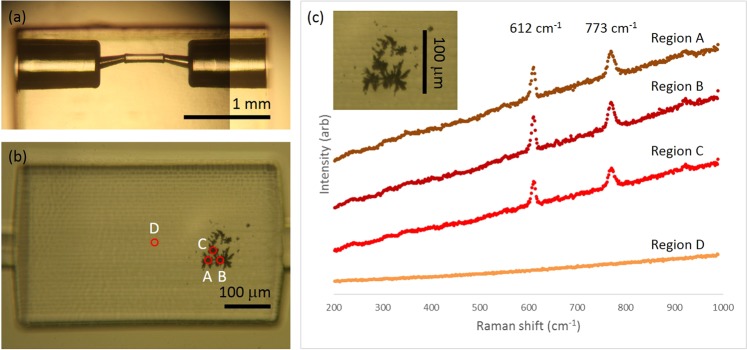
(**a**) Buried microchannel made using ultrafast laser inscription followed by selective chemical etching, showing inlet, outlet and SERS sensing region in the narrow centre (before the AgNP SERS sensor was fabricated), (**b**) AgNPs written within microchannel using 0.02 mm/s, 400 scans and 15 mW, (**c**) SERS spectra collected from 1 mM R6G from 4 regions within microchannel, using 2 mW of Raman excitation power at 514 nm. Region A, B and C were on the dark, AgNP region. Region D spectrum was collected approximately 100 *μ*m away from the AgNP area. Inset shows the AgNP region from (**b**).

Creating SERS surfaces *in situ*, within microfluidic devices, circumvents the need to pre-mix analyte with colloid, and offers controlled arrangements of AgNPs on the microfluidic surface with high sensitivity and large enhancements. Due to the nature of our laser fabrication method, there is scope to write these surfaces in buried microfluidics for high throughput, flow sensing of small molecules or cells. We have fabricated, using the same laser system as described above, a sub-surface microfluidic channel in fused silica and proceeded to write SERS substrates on an inside wall of the channel. To the best of our knowledge this is the first time that ultrafast laser machining of a subsurface channel has been combined with laser writing of AgNP SERS sensors. Preliminary results, showing Raman enhancement within femtosecond laser-inscribed monolithic devices, are presented in Fig. 7.

The buried microchannel in Fig. 7a is fabricated in fused silica using ultrafast laser inscription followed by selective chemical etching to etch out the inscribed volume following a previously published method^60^. After that, the channel is filled with the AgNP precursors as outlined above and a similar step to write the structure in Figs. 4d and 6a is followed, but with a power of 15 mW, since the microchannel surface is less smooth resulting in more scattering as compared to the top surface of fused silica. The silver structures can be seen as a dark patch around 100 *μ*m in diameter in Fig. 7b and inset Fig. 7c. The channel is emptied, rinsed in deionised water and 1 mM R6G is flowed through the channel. The Raman signal acquired from the AgNP surface from three locations where AgNPs are present (region A, B and C), and from a region approximately 100 *μ*m from the AgNPs patch, with no AgNPs (Region D), can be seen in Fig. 7c and can be seen in Supplementary Fig. S3.

An enhancement of the Raman signal is clearly observed. The average peak height for the three 612 cm^−1^ peaks from regions A-C on the microchannel wall was 3,482 counts +/− 11.90% and the height of the 773 cm^−1^ peak is 1857 counts +/− 19.39% thereby validating this approach (with scope for further optimization) for fabricating Ag SERS monitors within buried microfluidic channels in fused silica. The prospect of laser machining monolithic, microfluidic SERS devices for detecting trace amounts of substance in flowing liquid has potential for a wide array of bio and molecular sensing or characterisation experiments within bespoke three dimensional optofluidic or microfluidic devices.

Table 1, comparing techniques to generate SERS surfaces within microfluidic channels, is shown above to put the work presented in this manuscript into context.

**Table 1:** List of published work where SERS surfaces are integrated into microfluidic channel.

Author [reference] year	SERS fabrication method	Device material	Channel fabrication method	Analyte and minimum conc. measured or EF	SERS surface generated *in situ* (in channel)?
Mackenzie *et al*. [this paper]	Ultrafast laser photoreduction of Ag^+^	Fused silica	Ultrafast laser inscription then chemical etching	1 mM R6G (Rhodamine 6G) On surface AEF ~1 × 10^11^	yes
Liu *et al*.^61^	Ag thin film on nanowells in PDMS	glass	Bonding of glass and Ag film on textures PDMS	R6G EF 10^7^	no
Connatser *et al*.^62^	Electron beam lithography/Physical vapour deposition (PVD) of silver metal onto glass slab	PDMS	Soft lithography then glass bonding of SERS surface	70 nM Resorufin	no
Xu *et al*.^51^	Femtosecond direct laser writing onto channel	Glass	Microchannel made in glass using photolithography and wet etching techniques. Sealed with PDMS film after AgNPs written	*p*-aminothiophenol (*p*-ATP) EF 4 × 10^8^	no
Lee *et al*.^63^	Gold-patterned microarray and hollow gold nanospheres (HGNs)	PDMS	PDMS device made using soft lithography. Glass patterned with gold microarray wells, was fabricated by a photolithographic technique and bonded to PDMS	Alpha-fetoprotein (AFP) LOD 0–1 ng/mL	no
Parisi *et al*.^56^	*In situ* electrodeposition of copper in microfluidic channels. Cu(OAc)_2_ and CTAB flowed through the channel, Cu and C nanowalls formed on the working electrode, AgNO_3_ solution was flowed through, galvanic replacement of Cu with Ag+, formation of AgNPs.	PDMS	Electrodes integrated into the channel were pre-patterned on Si substrate using standard photolithography and lift-off techniques.	50 pM Crystal violet AEF 1.1 × 10^9^	yes, bonding step required first
Kim *et al*.^64^	Gold film-over nanospheres (AuFON)	PDMS	Soft lithography then glass bonding of SERS surface	1 nM BPE (trans-1,2-bis(4-pyridyl)ethylene)	no
Kim *et al*.^64^	Gold nanorods and nanocubes incorporated directly into the microfluidic polymer layer	PDMS	Soft lithography with AuNPs embedded in elastomer then glass bonding of SERS surface	1 mM BPE EF 4.1 × 10^7^	no
Oh & Jeong^65^	Thin silver film thermally evaporated onto channel surface	PDMS	Unwanted Ag film removed by scotch tape. Ag coated PDMS channel boded to glass	Benzenethiol EF 1.1 × 10^7^	no
Li *et al*.^66^	Nanoporous gold disks (NPGDs) fabricated on glass substrate using nanosphere lithography	PDMS	Bonding of glass containing NPGDs to PDMS	1 μm R6G EF 10^6^	no
Parisi *et al*.^67^	*In situ* galvanic replacement of a pre-patterned copper (Cu) substrate. Cu substrates pre-patterned on Si substrate using standard photolithography and lift-off techniques	PDMS	PDMS channel plasma bonded to the Si substrate containing the pre-patterned Cu substrates. AgO_3_ pumped through channel for galvanic replacement of pre-patterned Cu with Ag + until dense layer of AgNPs form.	Crystal violet AEF 2.2 × 10^7^	yes, bonding step required first
Novara *et al*.^68^	Electrochemical etching of a silicon wafer in HF electrolyte, followed by immersion plating in silver nitrate solution^69,69^	PDMS	Bonding of PDMS to Ag-coated porous silicon membranes (Ag-pSi)	4-Mercaptobenzoic acid (4-MBA) EF 10^7^	no

To the best of our knowledge, no reported method can inscribe SERS surfaces *in situ*, without requiring a bonding step. Further, using a laser inscription process very similar to the microfluidic channel inscription process, makes our straightforward ultrafast laser machining process suitable for rapid prototyping of complex microfluidic systems with functional SERS surfaces.

### Analytical estimate of the SERS enhancement factor for silver clusters

**Figure 8:**
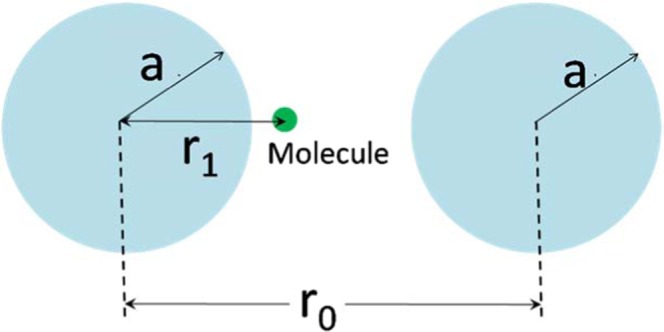
Geometry considered for estimation of SERS enhancement factor.

In order to compare the experimentally observed enhancement of Raman signal on our substrates with a theoretical estimate, we considered a simple geometry, namely two spherical nanoparticles with identical radius *a* separated by a distance of *r*_0_ as shown in Fig. 8.

A molecule which is placed at a distance *r*_1_ from one of the particles will experience an enhanced electric field due to coupling of their plasmon resonances. Following the treatment as described by Sun *et al*.^69^, the electric field enhancement factor *F* in the gap, considering only dipole-dipole coupling, can be obtained as,

5 $$F=\frac{{\rm{\omega }}}{\sqrt{2}}|\frac{1}{{m}_{11}+{m}_{12}}|({(\frac{a}{{r}_{1}})}^{3}+{(\frac{a}{{r}_{0}-{r}_{1}})}^{3})$$

Here, ω is the angular frequency of the incident radiation, *a*, *r*_0_ and *r*_1_ are the geometrical dimensions as shown in Fig. 8 and *m*_11_ and *m*_12_, the transfer matrix elements relating the electric field components, are given by,

6a $${m}_{11}=j({\rm{\omega }}-{{\rm{\omega }}}_{d})+\frac{{\rm{\gamma }}}{2}$$

6b $${m}_{12}=j{{\rm{\omega }}}_{d}{(\frac{a}{{r}_{0}})}^{3}$$

where γ in Eq. 6a is the damping factor of the free electrons in the metal layer. The dipole resonance frequency ω_*d*_ in Eqs. (6a) and (6b) is given, in terms of the plasma frequency ω_*p*_, of the nanoparticle material and the relative permittivity of the surrounding dielectric medium ${\epsilon }_{{\rm{D}}}$ as,

7 $${{\rm{\omega }}}_{d}={{\rm{\omega }}}_{p}\sqrt{\frac{1}{1+2{\epsilon }_{{\rm{D}}}}}$$

**Figure 9:**
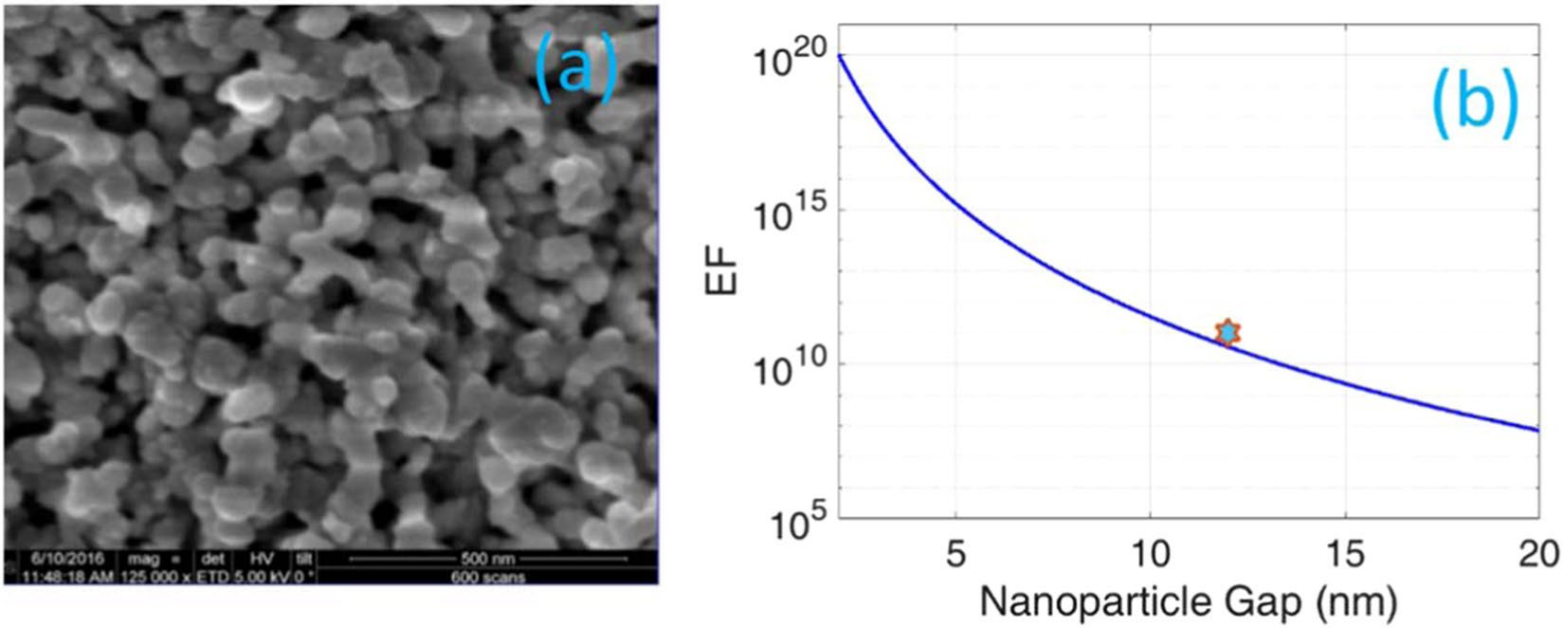
(**a**) SEM image (from 4 h) showing the typical size of AgNP clusters obtained from the multi-scan process, (**b**) The calculated Raman signal enhancement (EF) as a function of inter-particle gap for particle size of 50 nm corresponding to typical cluster size in (**a**). The data point corresponding to the experimentally obtained enhancement factor of 1.4 × 10^11^ is highlighted.

The plasma frequency of silver, which forms the nanostructures shown in Fig. 4h (replicated in Fig. 9a), resulting in SERS enhancement, is taken to be $\hslash {{\rm{\omega }}}_{{\rm{p}}}$ = 8.9 eV and surrounding medium is air with *∈*_*D*_ = 1. The typical size of the nanostructures shown in Fig. 4h (and Fig. 9a) is around 50 nm. Molecules will be separated from the nanostructures with a range of distances *r*_1_. The SERS enhancement factor which is *F*^4^, computed from Eq. (5) is plotted as a function of effective gap between two symmetric nanostructures as shown in Fig. 9b. The enhancement is computed for a molecule situated in the middle of the gap, i.e. ${r}_{1}={r}_{0}/2.$ As shown in Fig. 9b, enhancement factor increases with decreasing gap due to the formation of a “hot spot”^11^. The effective gap corresponding to the experimentally observed enhancement factor was about 10 nm implying that the experimental observation can be explained by considering silver clusters with a typical size of 50 nm and effective separation between Rhodamine 6G and the clusters being around 5 nm. Although we do not have the experimental resolution to precisely measure these geometric parameters with current data, they appear to be reasonably within the expectations from the cluster size distribution shown in Fig. 9a.

## Conclusions

Using laser photoreduction of silver cations we controllably write SERS AgNP surfaces on fused silica substrates. Single scans give us non-monotonic, non-reproducible results. Low scan speeds provided better surface coverage of silver nanoparticles. Further, multi-scan techniques gave good, uniform coverage of AgNPs and higher enhancement of Raman signals across the written surface, compared to single scan surfaces. A scan rate of 0.02 mm/s, a laser power of 5 mW and a scan number of 400 were found to be the optimal parameters for writing AgNP on fused silica, in this study. The typical size of AgNP clusters were found to be around 50 nm and the highest enhancement factors, of approximately 1.4 × 10^11^, obtained experimentally, are consistent with theoretical estimates for the observed AgNP size distribution. The remarkable advantage of our method is that the laser fabrication of the surfaces can be coupled with laser fabrication of buried microchannels, with enhancements in the Raman signal generated in a microflow of 1 mM R6G, which holds promise for developing monolithically integrated, tailored SERS microfluidic devices for small molecule or cell sensing, or point of care devices for rapid, specific and sensitive testing for analytes in fluid.

## Supplementary information

Supplementary Figures

Dataset 1

## Data Availability

The datasets generated during and/or analysed during the current study are available from the corresponding author on reasonable request.
