# Publisher Correction: Femtosecond laser fabrication of silver nanostructures on glass for surface enhanced Raman spectroscopy

**DOI:** 10.1038/s41598-020-59779-6

**Published:** 2020-03-16

**Authors:** Mark MacKenzie, Haonan Chi, Manoj Varma, Parama Pal, Ajoy Kar, Lynn Paterson

**Affiliations:** 1 Institute of Photonics and Quantum Sciences, School of Engineering and Physical Sciences, Heriot Watt University, Edinburgh, EH14 4AS UK; 2 Institute of Biological Chemistry, Biophysics and Bioengineering, School of Engineering and Physical Sciences, Heriot Watt University, Edinburgh, EH14 4AS UK; 3 Centre for Nano Science and Engineering (CeNSE), Indian Institute of Science, Bangalore, Karnataka India; 4 TCS Research and Innovation, Tata Consultancy Services, Bangalore, India

Correction to: *Scientific Reports* 10.1038/s41598-019-53328-6, published online 19 November 2019

The original version of this Article contained errors in the Reference list. Reference 69 was a repetition of reference 43.

In addition, Reference 55 was incorrectly given as:

Le, R. E. C., Blackie, E., Myer, M. & Etchegoin, P. G. Surface enhanced Raman scattering enhancement factors: A comprehensive study. *J. Phys. Chem. C* **111**, 13794–13803 (2007).

The correct reference is given below:

Le Ru, E. C., Blackie, E., Myer, M. & Etchegoin, P. G. Surface enhanced Raman scattering enhancement factors: A comprehensive study. *J. Phys. Chem. C* **111**, 13794–13803 (2007).

Furthermore, this Article contained typographical errors in the reference citations.

In the Introduction,

“This aspect, coupled with ultrafast laser inscription techniques for writing reproducible, sub-wavelength structures directly using femtosecond laser pulses by virtue of strong, highly localized nonlinear optical effects^55^, enables a unique approach for obtaining versatile SERS-active devices at high throughputs.”

now reads:

“This aspect, coupled with ultrafast laser inscription techniques for writing reproducible, sub-wavelength structures directly using femtosecond laser pulses by virtue of strong, highly localized nonlinear optical effects^58^, enables a unique approach for obtaining versatile SERS-active devices at high throughputs.”

In the Materials and Methods,

“Since we do not know the number of molecules probed and wish to know how much more signal is generated by SERS compared to normal Raman, the analytical enhancement factor (AEF) as described by Le Ru *et al*.^57^ (also known as the apparent enhancement factor)^58^ can be calculated using the following definition:”

now reads:

“Since we do not know the number of molecules probed and wish to know how much more signal is generated by SERS compared to normal Raman, the analytical enhancement factor (AEF) as described by Le Ru *et al*.^55^ (also known as the apparent enhancement factor)^57^ can be calculated using the following definition:”

In Table 1 Reference 57 was incorrectly given as Reference 56. Additionally, References 60–67 were incorrectly given as References 61–68 respectively.

Also in Table 1,

“Electrochemical etching of a silicon wafer in HF electrolyte, followed by immersion plating in silver nitrate solution^69,69^”

now reads:

“Electrochemical etching of a silicon wafer in HF electrolyte, followed by immersion plating in silver nitrate solution”

In the Results and Discussion section, under the subheading ‘Analytical estimate of the SERS enhancement factor for silver clusters’,

“A molecule which is placed at a distance *r*_1_ from one of the particles will experience an enhanced electric field due to coupling of their plasmon resonances. Following the treatment as described by Sun *et al*.^69^”

now reads:

“A molecule which is placed at a distance *r*_1_ from one of the particles will experience an enhanced electric field due to coupling of their plasmon resonances. Following the treatment as described by Sun *et al*.^68^”

This Article contained a typographical error in the Results and Discussion section, under the subheading ‘SERS-active buried channels’,

“Table 1, comparing techniques to generate SERS surfaces within microfluidic channels, is shown above to put the work presented in this manuscript into context.”

now reads:

“Table 1, comparing techniques to generate SERS surfaces within microfluidic channels, is shown below to put the work presented in this manuscript into context.”

These errors have been corrected in the PDF and HTML versions of the Article.

