# Supplementary material for: Femtosecond laser fabrication of silver nanostructures on glass for surface enhanced Raman spectroscopy
Source: Sci Rep. 2019 Nov 19;9:17058. doi: 10.1038/s41598-019-53328-6 (PMC6864074; doi:10.1038/s41598-019-53328-6)

**Femtosecond laser fabrication of silver nanostructures on glass for surface enhanced Raman spectroscopy**

Mark MacKenzie^1^, Haonan Chi^2^, Manoj Varma^3^, Parama Pal^4^, Ajoy Kar^1^, Lynn Paterson^2^.

1. Institute of Photonics and Quantum Sciences, School of Engineering and Physical Sciences, Heriot Watt University, Edinburgh EH14 4AS, UK.

2. Institute of Biological Chemistry, Biophysics and Bioengineering, School of Engineering and Physical Sciences, Heriot Watt University, Edinburgh EH14 4AS, UK.

3. Centre for Nano Science and Engineering (CeNSE), Indian Institute of Science, Bangalore, Karnataka, India.

4. TCS Research and Innovation, Tata Consultancy Services, India

**Supplementary figure S1**

SERS spectra from 5 regions (A-E) on AgNP surface (1 mM R6G, 514 nm laser, 2 mW laser power, 1 s accumulation) (See figure 5 in main article).


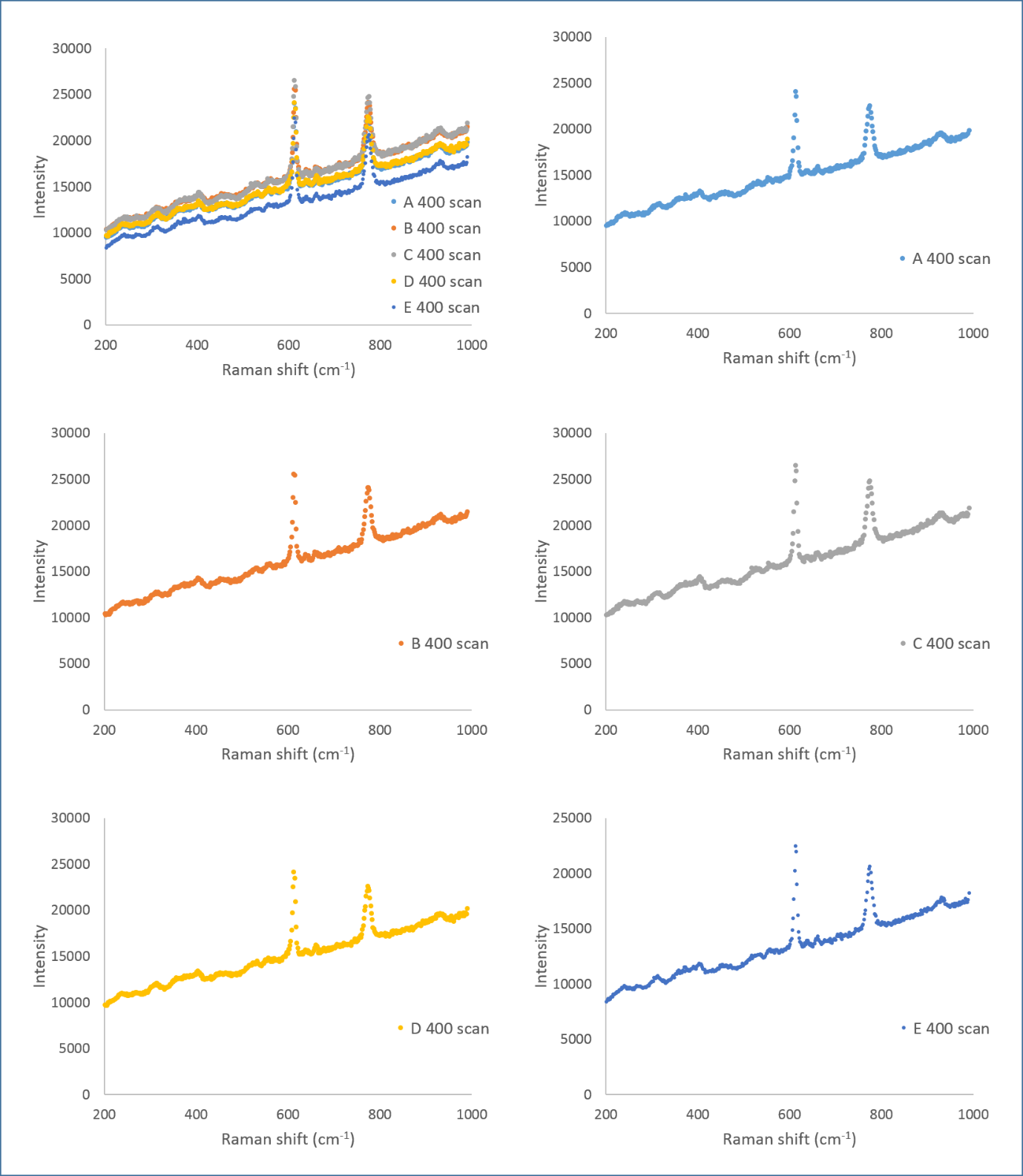


**Supplementary figure S2**

8 Non-SERS spectra (0.1 M Crystal Violet, 10 mW laser power, 514 nm laser, 1 second accumulation). (See figure 6 in main article).


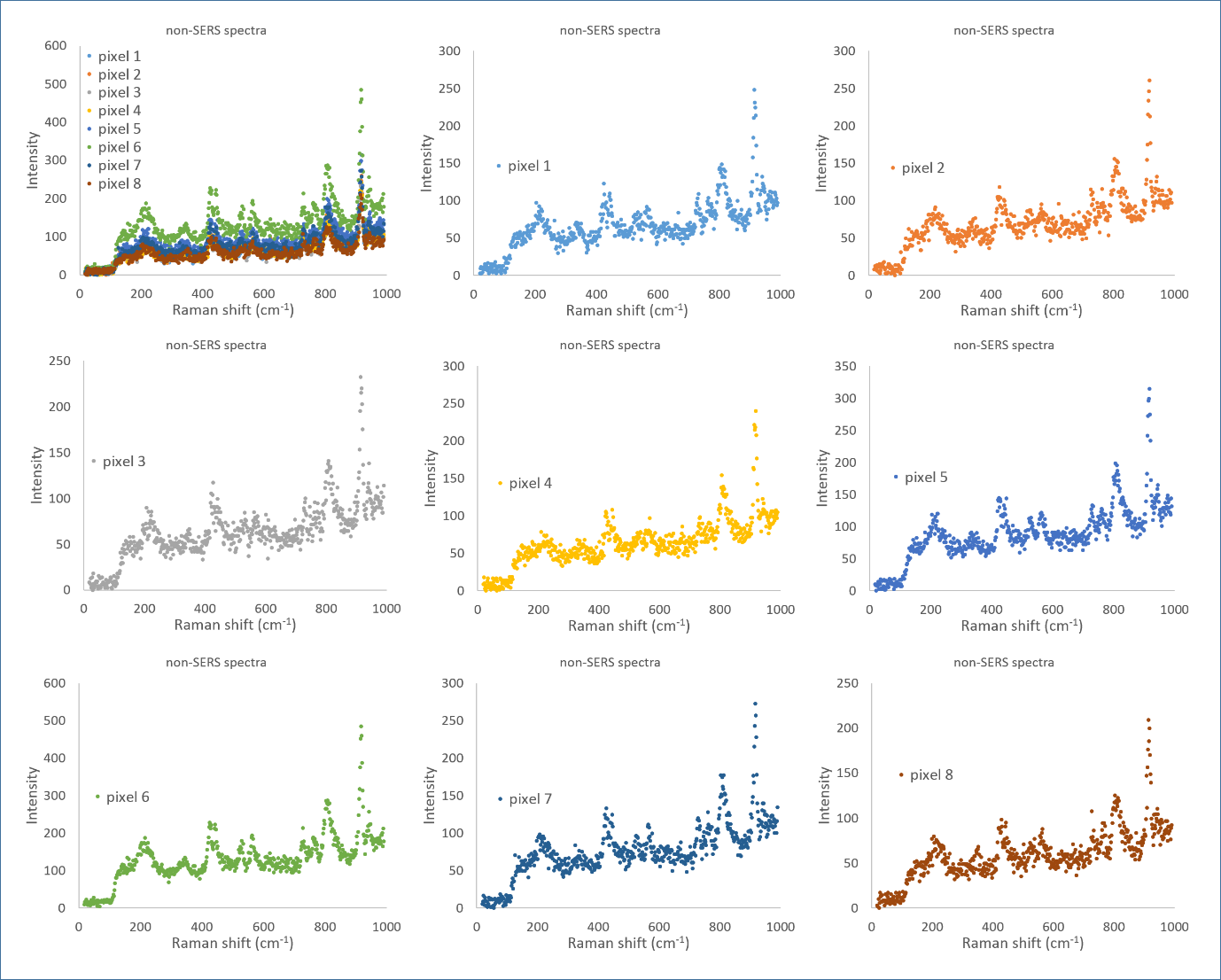


**Supplementary figure S3**

Spectra from four areas (A-D) within a sub-surface microfluidic channel (1 mM R6G, 514 nm laser, 2 mW laser power, 1 second accumulation). (See figure 7 in main article).


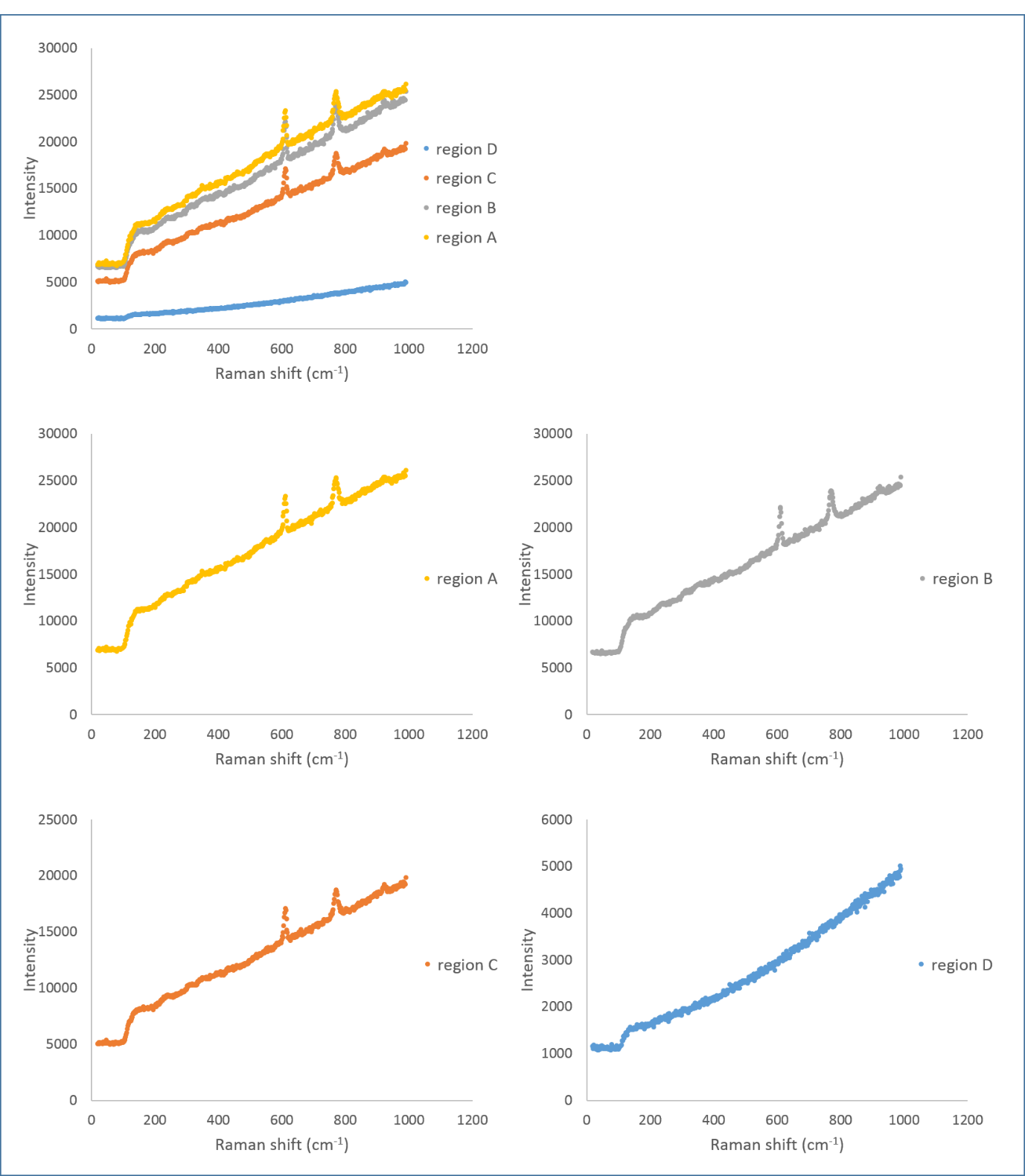

Supplement: Supplementary file 1 — Supplementary Figures [file 41598_2019_53328_MOESM1_ESM.docx]
